# Optimal Cutoff for the Neutrophil-to-Lymphocyte Ratio as a Tool for Pre-chemotherapy Prognosis Stratification of Breast Cancer Patients

**DOI:** 10.7759/cureus.90705

**Published:** 2025-08-21

**Authors:** Armita Zandi, Alyssa Qian, Maha Othman

**Affiliations:** 1 Department of Biomedical and Molecular Sciences, Queen's University, Kingston, Canada; 2 Department of Biomedical and Molecular Sciences, Queen's University, Kingston, CAN; 3 School of Baccalaureate Nursing, St. Lawrence College, Kingston, CAN; 4 Faculty of Medicine, Mansoura University, Mansoura, EGY

**Keywords:** biomarkers, blood cell count, chemotherapy, clinical oncology, inflammation

## Abstract

Introduction

The neutrophil-to-lymphocyte ratio (NLR) is an established inflammatory marker in cancer patients. The optimal cut-off as an independent prognostic factor for breast cancer (BC) progression in patients undergoing chemotherapy remains debatable, hindering effective stratification. This study explored the optimal NLR cut-off by comparing various thresholds and assessing their effectiveness in stratifying BC patients according to prognosis.

Methods

This was a longitudinal quantitative study conducted at Queen's University in Kingston, Ontario, Canada, and the associated hospital is Kingston General Hospital. Demographic, clinical, and cancer-specific data on 42 BC patients were recorded, including complete blood counts before and after two cycles of chemotherapy. The receiver operating characteristic curve assessed discriminatory performance. Diagnostic metrics and Youden’s J index were calculated, and McNemar’s test was used to compare baseline NLR cutoffs of 2.5, 3.0, and 3.5. Kaplan-Meier curves assessed the relationship between various NLR cut-offs and other cancer prognostic markers.

Results

The three NLR cutoffs demonstrated distinct diagnostic metrics and Youden’s J index values (p < 0.001), with the 3.0 cutoff providing the most balanced performance. Patients with pre-chemotherapy NLR > 3.0 were predicted to develop advanced stage BC more rapidly compared to those with pre-chemotherapy NLR < 3.0.

Conclusion

We believe that a more stringent NLR cutoff of 3.0 may be a suitable predictor of prognosis in BC patients based on the ranges evaluated in the literature. Findings of this paper could help clinicians in stratifying BC patients by risk, improving personalized treatment intensity while monitoring strategies accordingly.

## Introduction

The neutrophil-to-lymphocyte ratio (NLR) is calculated from peripheral blood counts [1]. This value reflects the balance between the innate, represented by neutrophil, and adaptive, represented by lymphocyte, immune responses [1]. In healthy individuals, NLR typically ranges between 1 and 2, while values >3 or <0.7 may indicate pathology [2,3]. Elevated NLR may result from increased neutrophil counts and/or decreased lymphocyte counts. These changes commonly are observed in conditions involving systemic inflammation, such as bacterial infections [4], stroke [5], post-surgical complications [6], and cancer [2,4,7]. In such settings, the inflammatory response often suppresses neutrophil apoptosis and impairs adaptive immunity, contributing to disease progression [2].

In oncology, a higher NLR is generally associated with poorer prognosis [8]. Specifically, a range of 2.3-3.0 suggests early warnings of poorer survival or higher rates of recurrence [8]. For example, in stage IV non-small cell lung cancer, elevated baseline NLR (>4.95) has been linked to more brain metastases [6]. Moreover, in epithelial ovarian cancer, high pre-treatment NLR independently predicted reduced overall survival [7]. In breast cancer (BC), NLR has been proposed as a cost-effective prognostic biomarker, but findings remain inconsistent [9-12]. Some studies have reported significant associations between elevated NLR and reduced disease-free and overall survival [10], while others found mixed or no prognostic value, particularly in early-stage disease or patients receiving neoadjuvant chemotherapy (NACT), administered before surgery to shrink the tumor [11,12]. 

BC remains the second leading cause of cancer death in women [9]. Prognosis depends on both patient-specific and tumor-related factors, including stage, grade, molecular subtype, metastases, and receptor status [9]. NACT, commonly used in high-risk subtypes such as triple-negative breast cancer (TNBC), is associated with acute and prolonged systemic inflammation, potentially influencing prognosis [13-15]. Given its potential link to cancer biology and treatment-related inflammation, NLR could help stratify patients and guide management, particularly when combined with established prognostic indicators.

However, uncertainty remains regarding the optimal NLR cutoff for BC prognosis and whether NLR functions as an independent prognostic marker beyond established factors. This study evaluated the prognostic value of varying pre-chemotherapy NLR thresholds in BC, both as independent and dependent prognostic markers. In addition, we hope to identify a more informative cutoff range. Finally, we aim to explore NLR changes during chemotherapy to explore its potential role in patient monitoring. 

This article was published previously as a preprint on Research Square on 02/27/2025.

## Materials and methods

Patient recruitment and data collection

This longitudinal quantitative study is a part of an ongoing study where BC patients are recruited prior to their first chemotherapy treatment and are followed throughout their treatment process. It was conducted at Queen's University in Kingston, Ontario, Canada, and the associated hospital is Kingston General Hospital. Forty-two female breast cancer patients scheduled for neoadjuvant chemotherapy were recruited between January 2022 and January 2024. Additional inclusion criteria were a life expectancy of at least three months or more, and participants must have had no existing use of anticoagulants to avoid affecting neutrophil counts. Baseline demographic data, including age, weight, body mass index (BMI), and comorbidities, were collected from the participants with complete blood counts (CBC) conducted prior to and after two cycles of chemotherapy. NLR was calculated for all participants prior to the start of chemotherapy and after the first and second cycles. Additional cancer-specific data, such as cancer stage, lymphovascular invasion, and the receptor information, including estrogen receptor (ER), progesterone receptor (PR), and human epidermal growth factor receptor 2 (HER-2) status, were also recorded.

Statistical analysis

Descriptive statistics (mean, median, mode, standard deviation), percentages, and frequency were used to analyze patients’ and cancer-specific characteristics. A Shapiro-Wilk test was conducted and concluded that the data collected were not normally distributed (p < 0.001). Hence, non-parametric equivalents of statistical tests were employed. Receiver operating characteristic (ROC) curves were generated for each diagnostic test (various NLR cutoffs) and sub-groups (those who received immunotherapy vs. those who did not) to assess their discriminatory performance. From these curves, sensitivity, specificity, accuracy, positive predictive value (PPV), negative predictive value (NPV), and Youden’s J index were calculated to characterize test performance comprehensively. In addition, McNemar’s test was performed to statistically compare the selected cutoffs within the same patient cohort, ensuring that differences in diagnostic performance metrics were rigorously evaluated. Furthermore, Kaplan-Meier curves were generated to assess and compare the cancer prognostic factors when baseline NLR was above or below 3.0, evaluating the potential of NLR as a BC prognostic factor. Covariates were adjusted, and the log-rank was used to compare the curves. IBM SPSS Statistics for Windows, version 29 (released 2022, IBM Corp., Armonk, NY) was used for all statistical analyses, with a p-value less than 0.05 indicating statistical significance.

## Results

Patient cohort

A total of 42 breast cancer patients (age: mean (M) = 57.9, standard deviation (SD) = 11.9, range (R) = 34.0-85.0; BMI: M = 28.7, SD = 8.7, 20.0-58.6) were examined. Twenty-three patients had early-stage cancer (stages I and II), and 19 had advanced stages (III and IV). In addition, the average NLR before chemotherapy was M = 2.8 (SD = 1.3, 0.9-12.2), after Cycle 1 was M = 4.7 (SD = 5.1, 0.8-27.8), and after Cycle 2 was M = 7.5 (SD = 7.7, 1.0-31.5) (Table 1). Pre-chemotherapy NLR was negatively correlated with patient age (r = -0.19, p = 0.12) and positively correlated with BMI (r = -0.08, p = 0.30), cancer staging (r = 0.09, p = 0.30), lymphovascular invasion (r = 0.11, p = 0.25), PR negative (r = 0.08, p = 0.30), and HER2 negative (r = 0.05, p = 0.38). Statistically significant negative correlations were seen between pre-chemotherapy NLR and ER-negative (r = 0.30, p = 0.029) and triple-negative breast cancer (TNBC) subtype (r = 0.37, p = 0.008). The TNBC subtype has been shown in the literature to have a worse prognosis compared to other breast cancer subtypes. This significant relationship between pre-chemotherapy NLR and TNBC suggests a potential link between NLR and overall prognosis, highlighting its possible prognostic value.

**Table 1:** Patient demographics and cancer-specific data. Patient demographic includes age, body mass index (BMI), baseline (pre-chemotherapy) NLR, NLR after cycle 1 chemotherapy, and NLR after cycle 2 chemotherapy. Cancer-specific data includes cancer stages (I-IV), presence of metastasis, expression of estrogen receptors (ER), progesterone receptors (PR), HER2 receptors, and triple-negative breast cancer. The absolute frequency of patient data is expressed in N and percentage (%).

	Total (n)	Percentage (%)
Age (years)		
≤40	4	9.52
41-50	8	19.1
51-60	11	26.2
61-70	12	28.6
71-80	6	14.3
≥ 80	1	2.4
BMI		
≤20.00	1	2.4
20.01-25.00	16	38.1
25.01-30.00	12	28.6
30.01-35.00	6	14.3
35.01-40.00	3	7.1
≥40.01	4	9.5
Cancer stage		
I	13	31.0
II	10	23.8
III	9	21.4
IV	10	23.8
Metastasis		
No	31	73.8
Yes	8	19.1
Missing	3	7.1
Baseline/pre-chemotherapy NLR		
≤2.00	18	42.9
2.01-3.00	13	31.0
3.01-4.00	4	9.5
4.01-5.00	4	9.5
5.01-6.00	0	0.0
≥6.01	3	7.1
NLR after chemotherapy cycle 1		
≤2.00	6	14.3
2.01-3.00	7	16.7
3.01-4.00	5	11.9
4.01-5.00	4	9.5
5.01-6.00	2	4.8
6.01-7.00	1	2.4
≥7.01	4	9.5
Missing	13	31.0
NLR after chemotherapy cycle 2		
≤2.00	4	9.5
2.01-3.00	1	2.4
3.01-4.00	4	9.5
4.01-5.00	2	4.8
5.01-6.00	0	0.0
6.01-7.00	0	0.0
7.01-8.00	1	2.4
8.01-9.00	0	0.0
9.01-10.00	1	2.4
10.01-11.00	1	2.4
11.01-12.00	1	2.4
≥12.01	3	7.1
Missing	24	57.1
Estrogen receptor (ER)		
Positive (ER+)	25	59.5
Negative (ER-)	15	35.8
Missing	2	4.8
Progesterone receptor (PR)		
Positive (PR+)	18	42.9
Negative (PR-)	23	54.8
Missing	1	2.4
Human epidermal growth factor receptor 2 (HER2)		
Positive (HER2+)	17	40.5
Negative (HER2-)	24	57.1
Missing	1	2.4
Triple-negative		
Triple-negative	11	26.2
Non-triple-negative	31	73.8

Comparison of various NLR cutoffs regarding their effectiveness in BC prognosis

The most clinically relevant pre-chemotherapy NLR cutoff, which, according to the literature, is 2.3 to 3 [9]. Expanding from this value, potential NLR cutoffs include 2.5 (close to the median of baseline NLR), 3, and 3.5. Although 3.5 exceeded the range suggested by the literature, we wanted to explore whether the range could be expanded upwards. In addition, since the mean value of baseline NLR in our study population was close to 3.5, we hoped to assess this value to be more comprehensive. As summarized in Table 1, the sensitivity of the baseline NLR cutoffs of 2.5, 3.0, and 3.5 was 90.5%, 33.3%, and 57.1%, respectively, with corresponding specificities of 11.4%, 80.0%, and 34.3%. At the 2.5 cutoff, the accuracy, PPV, NPV, and Youden’s J index were 41.1%, 38.0%, 66.7%, and 0.019, respectively (Table 2). For the 3.0 cutoff, these values were 62.5%, 50.0%, 66.7%, and 0.133, and for the 3.5 cutoff, they were 42.9%, 34.3%, 57.1%, and -0.086, respectively (Table 2). McNemar’s test demonstrated statistically significant differences in the diagnostic performance across all three cutoff values (p < 0.001 for all comparisons).

**Table 2:** Performance metrics at various neutrophil-to-lymphocyte ratio (NLR) cutoff values for predicting advanced stage (stages III and IV) breast cancer.

Baseline NLR cutoff	2.5 cut off value	3.0 cut off value	3.5 cut off value
True positive (TP)	19	7	12
True negative (TN)	4	28	12
False positive (FP)	31	7	23
False negative (FN)	2	14	9
Sensitivity	90.5%	33.3%	57.1%
Specificity	11.4%	80.0%	34.3%
Accuracy	41.1%	62.5%	42.9%
PPV	38.0%	50.0%	34.3%
NPV	66.7%	66.7%	57.1%
Youden’s J index	0.019	0.133	-0.086

**Figure 1:**
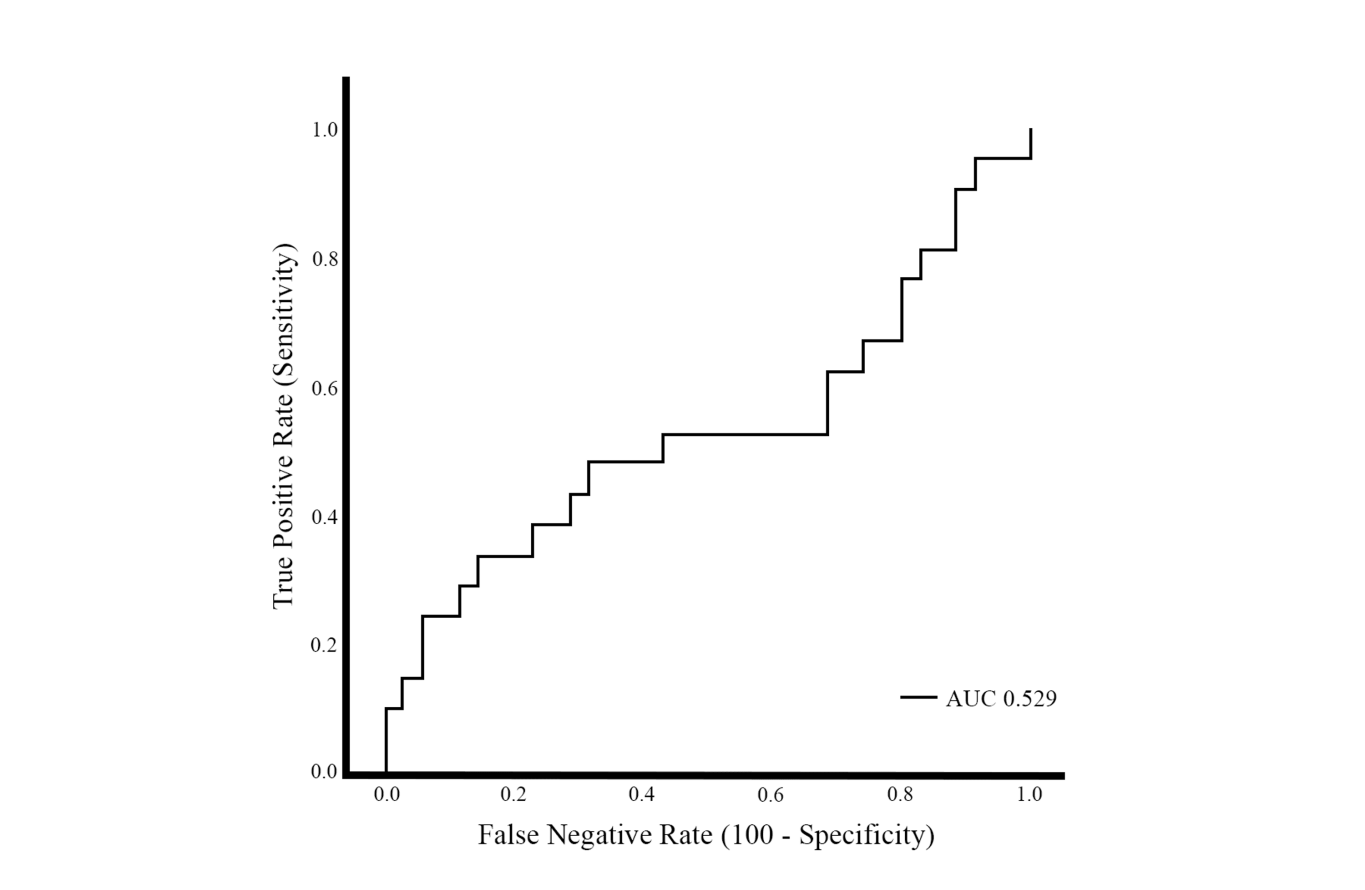
Receiver operating characteristic (ROC) curve evaluating the diagnostic performance of the neutrophil-to-lymphocyte ratio (NLR)-based predictive model. The curve plots sensitivity against 1−specificity at various cutoff values. The area under the curve (AUC) was 0.529.

Differential prognosis in groups of low and high NLR

**Figure 2:**
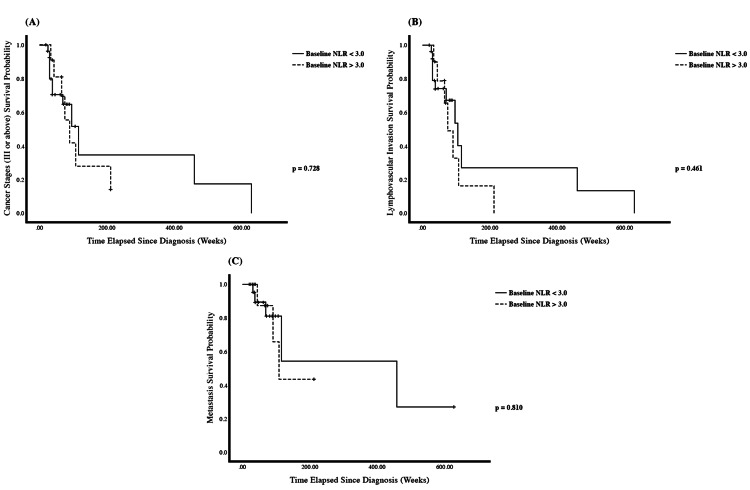
Kaplan–Meier estimate stratified according to pre-chemotherapy neutrophil-to-lymphocyte ratios (NLRs) above or below 3.0 in patients with breast cancer. (A) Cancer stage (III or IV) (p = 0.73). (B) Lymphovascular invasion (p = 0.46). (C) Metastasis (p = 0.81). +: Censored. Data points are censored if events (cancer stages, lymphovascular invasion, tumor grade, or metastasis) were not seen in the subject prior to the termination of this study (January 18, 2024). p-value <0.05 is considered statistically significant.

We then investigated the differences in breast cancer prognostic factors between patients’ groups with low (<3.0) and high (>3.0) pre-chemotherapy NLRs. There was a significant difference in the presence of ER-negative (p = 0.038) and TNBC (p = 0.014) between the two patient groups. However, the difference in cancer staging, lymphovascular invasion, metastasis, and presence of PR and HER2 receptors was not significant. In addition, according to the Kaplan-Meier analysis (Figure 2), patients with a pre-chemotherapy NLR < 3 had an estimated median progression-free time approximately 400 weeks longer than those with NLR ≥ 3, supporting the association between high NLR and adverse cancer-specific prognostic markers such as advanced stage, lymphovascular invasion, and metastasis.

**Figure 3:**
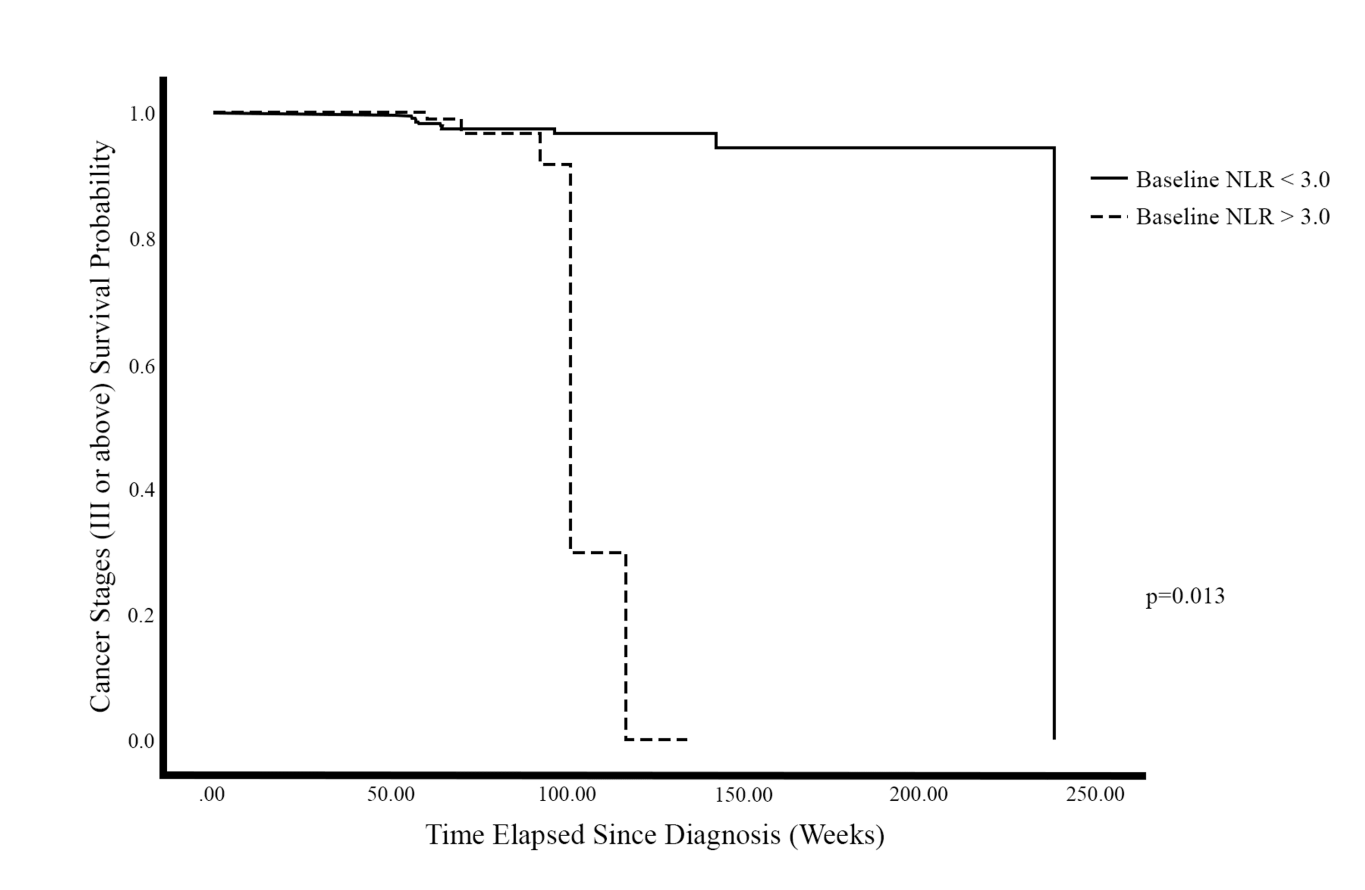
Cox regression survival analysis of advanced cancer stage development according to pre-chemotherapy NLR, ER-negative, and TNBC stratifications. p-value <0.05 is considered statistically significant. NLR: neutrophil-to-lymphocyte ratio, ER: estrogen receptor, TNBC: triple-negative breast cancer

We further examined NLR as a dependent predictive biomarker for advanced cancer stages alongside ER-negative and TNBC. Figure 3 shows that pre-chemotherapy NLR is a significant predictive biomarker (p = 0.013). Patients with high pre-chemotherapy NLR combined with ER-negative or TNBC were estimated to develop advanced cancer stages approximately 100 weeks sooner than those with lower pre-chemotherapy NLRs (Figure 3).

## Discussion

This study revisited the use of NLR, an established marker of inflammation and a previously reported cancer prognostic marker. It provided more analyses concerning clinically relevant pre-chemotherapy NLR cutoff levels. In addition, NLR changes following chemotherapy were assessed. Pre-chemotherapy NLRs exceeding 3.0 were found to be a strong independent predictor of poor BC outcomes.

Our positive correlation data reflect NLR sensitivity to hormone receptor expressions (ER-negative and TNBC), which was confirmed in a recent meta-analysis involving 2,069 patients from 10 studies [16]. The negative correlation of NLR with patients’ age can be explained by the decline in neutrophil counts and elevation of lymphocyte counts as one ages [17]. We have hypothesized the following possibilities. Studies have found that neutrophil proliferation response was less sensitive among older adults [17]. Another plausible consideration is menopause, although there have been mixed findings reported on the effect of menopause on neutrophil and lymphocyte counts. Some have reported no significant difference in neutrophil counts between pre- and postmenopausal women, while others found reduced neutrophil counts and elevated lymphocyte counts in postmenopausal individuals [18,19]. Specifically, regarding NLR, the current literature suggests no significant associations between NLR and menopause, except when postmenopausal women have osteoporosis [19,20]. It is important to remember that the assessment of NLR in early-stage breast cancer presents significant methodological challenges that extend beyond postmenopausal considerations. Hence, a more comprehensive exploration of confounding variables is necessary before drawing definitive conclusions. Lastly, the positive correlation between high NLR and other cancer prognosis markers may suggest the association with high risks of a more aggressive subtype of breast cancer, such as TNBC.

This study concluded an NLR cutoff of 3.0 as a strong predictor of poor breast cancer outcomes, particularly when combined with other known prognostic factors, TNBC, or ER-negative BC. Furthermore, our detailed data on the various cut-off levels and the comparative analysis of patients with NLR above and below 3 have indicated that the stratification of patients’ assessment is possible. We, as others, believe that with CBC being a routine evaluation in cancer follow-up clinics, NLR could be a cost-efficient and convenient prognostic marker, especially if combined with other prognostic cancer-specific factors. This can help clinicians accurately and effectively assess patients throughout therapy.

Among the three evaluated cutoffs, 3.0 provided the best balance between sensitivity, specificity, and Youden’s J index. A cutoff of 2.5 achieved the highest sensitivity (90.5%) but very low specificity (11.4%), resulting in many false positives and a low Youden’s J index (0.019). Conversely, 3.5 had moderate sensitivity (57.1%) and low specificity (34.3%), yielding a negative Youden’s J (-0.086). Given these limitations, NLR should not be applied in isolation; integrating it with patient characteristics (e.g., age, comorbidities) or complementary biomarkers may improve predictive accuracy. Future studies should explore multi-parameter prognostic models to better stratify risk and guide individualized management.

By contrast, the 3.0 cutoff achieved a specificity of 80.0% and a sensitivity of 33.3%, resulting in the highest Youden’s J index (0.133) and overall accuracy (62.5%) among the three cutoffs. While this represents the best balance observed in our analysis, the limited sensitivity restricts its value as a standalone diagnostic tool. NLR should instead be considered within a broader prognostic framework, integrated with other clinical, pathological, or biomarker data to improve predictive accuracy.

This study has several limitations, foremost being the small sample size, which limits statistical power and generalizability. Survival information was not available, preventing evaluation of NLR’s association with overall and recurrence-free survival. Although comorbidity data were available, the sample size limited our ability to control for conditions known to influence NLR (e.g., chronic inflammatory diseases), which may have affected the observed sensitivity and specificity. In addition, our analysis aggregated all tumor subtypes without stratification; given the biological heterogeneity of breast cancer, NLR’s prognostic value may differ across molecular classifications (e.g., luminal A, luminal B, HER2-enriched, triple-negative). These results should be regarded as exploratory and hypothesis-generating, and future studies with larger, well-stratified cohorts are essential to confirm NLR’s prognostic value and refine cutoff thresholds for clinical use.

## Conclusions

This study re-examined NLR, an established marker of inflammation, for its potential in stratifying breast cancer patients and predicting prognosis. Our findings suggest that a cutoff of 3.0 may offer the most balanced performance in this context. We also observed that NLR increased throughout chemotherapy and was associated with ER-negative status, TNBC, and chemotherapy progression. Given the limitations of NLR as a standalone measure, it should be considered alongside other clinical, pathological, and biomarker data to enhance prognostic accuracy. Further validation in larger, well-stratified cohorts is warranted to confirm these findings and explore how NLR can contribute, in combination with other markers, to optimizing breast cancer care.
